# Comparative efficacy of medicaments or techniques for pulpotomy of primary molars: a network meta-analysis

**DOI:** 10.1007/s00784-022-04830-1

**Published:** 2022-12-29

**Authors:** Jiehua Guo, Na Zhang, Yuzhao Cheng

**Affiliations:** Department of Oral Medicine, Shanxi Provincial People’s Hospital, NO.29 The Twin Towers Temple District, Taiyuan, Shanxi China

**Keywords:** Primary molars, Pulpotomy, Pulpal medicaments, Mineral trioxide aggregate, Network meta-analysis

## Abstract

**Objectives:**

We performed this network meta-analysis to determine the comparative efficacy of formocresol (FC), ferric sulfate (FS), sodium hypochlorite (NaOCl), calcium hydroxide (CH), mineral trioxide aggregate (MTA), biodentine, and laser for pulpotomy of molar teeth.

**Materials and methods:**

An updated search was conducted in PubMed, Embase, and the Cochrane Library to identify relevant randomized controlled trials (RCTs) published before October 30, 2022, after screening previous meta-analyses. The Cochrane risk of bias assessment tool was used to appraise the methodological quality of included studies. Clinical and radiographic success rates were assessed as outcomes. Random network meta-analysis was performed by using STATA software (version 14.0) with “network” command.

**Results:**

A total of 43 RCTs were included. Network meta-analysis indicated that CH was inferior to other medicaments and techniques in all outcomes, and MTA and biodentine was better than FC, FS, and NaOCl in terms of clinical and radiographic success rates. Results of ranking probabilities suggested that MTA ranked first in all outcomes except for clinical success at both 6 months.

**Conclusions:**

Our results suggested that MTA was associated with significant improvement in both clinical and radiographic success than other pulpotomy medicaments and techniques, with the highest probability of being the optimal option.

**Clinical relevance:**

The current network meta-analysis determined the comparative efficacy and safety of 7 common pulpotomy medicaments in molar pulpotomy, including FC, FS, NaOCl, CH, MTA, biodentine, and laser, and the pooled results revealed comparable efficacy in clinical and radiographic success rates at 6 and 12 months between FC, FS, and NaOCl in primary molars pulpotomies. However, MTA, biodentine and laser may have more advantages than other pulpotomy medicaments for clinical and radiographic success. Therefore, in clinical practice, practitioners should select MTA, biodentine, or laser as pulpotomy medicaments in molar pulpotomy.

**Supplementary Information:**

The online version contains supplementary material available at 10.1007/s00784-022-04830-1.

## Introduction

Pulpotomy has been regarded as the most common treatment modality for carious pulp-exposed and symptom-free primary molars [1], which was performed to remove the inflamed or infected coronal pulp tissue and cover the pulp stumps with a therapeutic agent. Therefore, pulpotomy has significant advantages in preserving the vitality of the radicular pulp, limiting pain and inflammation, and maintaining involved tooth to the normal exfoliation stage [2, 3]. During pulpotomy treatment, it is critically important to appropriately select medicaments and techniques. According to Fuks et al. [4, 5], an ideal pulpotomy medicament and technique should be bactericidal, remain harmless to the pulp and surrounding structures, promote healing of the radicular pulp, and should not get involved with physiologic root resorption.

Several pulpotomy medicaments and techniques have been developed and used in primary molars pulpotomies, such as formocresol (FC), ferric sulfate (FS), sodium hypochlorite (NaOCl), calcium hydroxide (CH), and mineral trioxide aggregate (MTA) [6, 7]. FC, which was firstly introduced by Buckley in 1904 [8], is the most commonly used medicament in pulpotomy and has been regarded as a “gold standard” control in trials [7]. As a common haemostatic agent, FS has also commonly used for molar pulpotomy because it can create a shallow protective iron-protein layer complex through reacting with the pulp tissue upon its coverage [9]. Since the first application in endodontics as an irrigating agent, NaOCl has already been frequently used in molar pulpotomy because it has excellent antimicrobial and tissue dissolving properties. Moreover, the efficacy of biodentine and laser in molar pulpotomy as medicament and technique has also been discussed [10]. Although numerous studies have compared the efficacy of different pulpotomy medicaments and techniques, and MTA, FC, and biodentine have been recommended as pulpotomy medicaments by guidelines [11, 12], there are conflicting results about the pulpotomy medicaments and techniques.

Currently, several meta-analyses [13–15] have investigated the comparative efficacy of the different pulpotomy medicaments and techniques. For example, three meta-analyses investigated the effectiveness of FC and FS as pulpotomy material in primary molars [16–18]. One meta-analysis investigated the comparative efficacy of FS with other pulpotomy medicaments in primary molars [19], and another one investigated the comparative efficacy of FC, FS, MTA, CH, and lasers [20]. More importantly, Tewari et al. recently performed an overview of pairwise systematic reviews to determine the success of medicaments and techniques for pulpotomy and highlighted the lack of evidence regarding the choice of pulpotomy agents for caries-affected primary teeth [21]. We therefore performed the present network meta-analysis to determine the comparative success rate of eight medicaments and techniques including FC, FS, NaOCl, CH, MTA, biodentine, and laser for pulpotomy of primary molars in order to provide definitive evidence-based recommendations for clinical decision-making.

## Materials and methods

### Study design

The present study was a network meta-analysis of published randomized controlled trials (RCTs) [22], and therefore institutional ethical approval and patient’s informed consent were not required. The final results of this network meta-analysis were reported according to the Preferred Reporting Items for Systematic Reviews and Meta-Analyses (PRISMA) extension statement for reporting systematic reviews incorporating network meta-analysis (PRISMA-NMA) [23]. The present study protocol was not registered on a public platform.

### Data sources and search strategy

We searched PubMed, Embase, and the Cochrane library from their inception through October 30, 2022, for the identification of relevant RCTs. Several terms and their analogs were used to construct search strategy with Boolean operator, including “primary molars,” “pulpotomy,” “formocresol,” “ferric sulfate,” “sodium hypochlorite,” “calcium hydroxide,” “MTA,” “mineral trioxide aggregate,” “biodentine,” “laser,” and “randomized controlled trial.” Details of search strategies for PubMed, Embase, and the Cochrane library are shown in Table S1. Additional studies were identified through screening the reference lists of included studies and evaluating eligible studies of previous meta-analyses. Any discrepancies about study retrieval and selection between two authors were resolved based on the consensus principle.

### Study selection

Study selection was performed by two independent authors according to three steps as follows: (a) removal of duplicate studies based on EndNote X9, (b) initial eligibility evaluation through reviewing the titles and abstracts, and (c) final eligibility evaluation through screening the full texts. Any discrepancies were resolved based on the consensus principle between two authors.

### Inclusion criteria

According to the previous meta-analyses [18, 19], studies were considered to be eligible if they met the following criteria: (a) patients undergoing pulpotomy in vital primary molars with pulp exposure due to cries; (b) RCTs that evaluated the efficacy of FC, FS, NaOCl, CH, MTA, biodentine, and laser as a pulpotomy medicament and had zinc oxide-eugenol (ZOE) or reinforced ZOE (RZOE) as immediate restoration but amalgam or stainless steel crown as final restoration in vital primary molars, with a minimum follow-up time of 6 months; and (c) both clinical and radiographic success rates at 6 and 12 months were considered as outcomes. Studies were excluded if they met exclusion criteria as follows: (a) ineligible study design, such as animal study and single arm clinical trials; (b) no essential data for outcomes; and (c) duplicate studies with poor methodological quality and without adequate data.

### Data extraction

Two independent authors used predesigned data extraction sheet to extract the following essential data from the included studies: (a) general information of the included studies including the first author’s name, country, publication year, follow-up period; (b) basic characteristics of patients including the number of patients and teeth, mean age of patients, details of comparisons, and methods of isolation and restoration; (c) outcomes of interest including clinical and radiographic success rates at 6 and 12 months; and (d) details of methodology including 7 items proposed by the Cochrane risk of bias assessment tool [24]. The corresponding authors were contacted through email if essential data were missed from the included studies. Any discrepancies were resolved based on the consensus principle between two authors.

### Definition of outcomes

We evaluated clinical and radiographic success rates at 6 and 12 months. Clinical success was obtained if patients were absence of the symptoms of pain, tenderness to percussion, swelling, sinus opening, pathologic mobility, and radiographic success was demonstrated if periapical or furcal radiolucency, internal or external root resorption, loss of lamina dura, and pulp canal obliteration were not detected [18].

### Geometry of the network

Network plot was produced to illustrate the evidence structure of different medicaments for individual outcome. Node and line were the essential elements of evaluating geometry of the evidence network [25]. For this network meta-analysis, a node was used to represent individual pulpotomy medicament, and solid line directly connecting two independent nodes indicated the presence of direct comparison between two pulpotomy medicaments. Furthermore, the size of individual node was proportional to the accumulated number of eligible studies, and the thickness of a solid line was proportional to the accumulated number of direct comparisons between two pulpotomy medicaments.

### Risk of bias within study

Cochrane risk of bias assessment tool [24] was used to assess the methodological quality of the included studies. A value of “high,” “unclear,” or “low” was assigned according to seven items as follows: random sequence generation, allocation concealment, blinding of participant and personnel, blinding of outcome assessors, incomplete outcome data, selective outcome reporting, and other sources. The overall methodological quality of individual study was rated as “high,” “moderate,” or “low” according to the following criteria: (a) individual study was rated as “high” level if all items were labeled with “low” risk of bias, (b) individual study was rated as “moderate” level if at least one of all items was labeled with “unclear” risk of bias but no item was labeled with “high” risk of bias, and (c) individual study was rated as “low” level if at least one of all items was labeled with “high” risk of bias [26]. Any discrepancies were resolved based on the consensus principle between two authors.

### Statistical analysis

We performed random-effects network meta-analysis using STATA 14.0 (StataCorp LP, College Station, Texas, USA) with “network” command [27] after the assumption for homogeneity of the included studies and transitivity was established [28]. First, the global inconsistency was assessed using the design-by-treatment interaction model [29, 30], and the local inconsistency was assessed using the side-splitting strategy [31]. Meanwhile, loop inconsistency also assessed using the method described by Lu and Ades [32]. All estimates were expressed using odds ratio (OR) and the corresponding 95% confidence interval (CI). To facilitate the interpretation of the estimated treatment effects, we sued the surface under the cumulative ranking (SUCRA) to calculate ranking probability, and a higher the SUCRA value indicates a greater the probability of becoming better option [33]. For publication bias, we generated comparison-adjusted funnel plots [34, 35] for individual outcome, and a symmetric plot indicated the absence of publication bias. Graphical tools developed by Chaimani et al. [25] were sued to visualize all pooled results.

## Results

### Study retrieval and selection

**Fig. 1:**
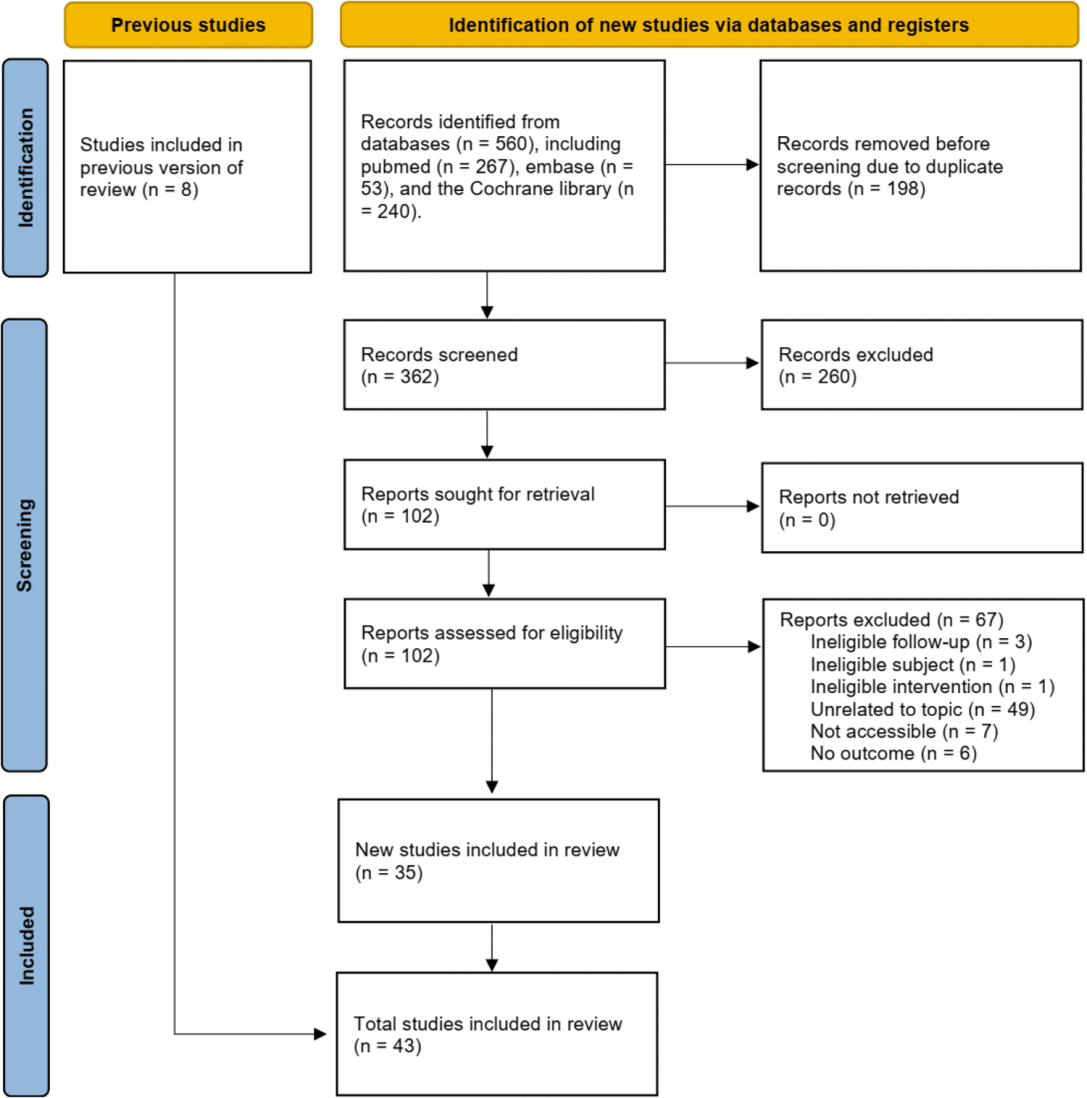
PRISMA flow diagram of study retrieval and selection

A total of 560 relevant studies were identified from our initial search. After removing 198 duplicate studies, 362 studies were retained for initial eligibility evaluation. Next, a total of 260 ineligible studies were excluded after screening the titles and abstracts. Full texts of 102 studies were retrieved for final eligibility evaluation because 8 eligible studies were identified from previous meta-analyses. Finally, 43 studies [2, 3, 10, 36–75] were included in the network meta-analysis after excluding 66 studies due to ineligible follow-up period (*n* = 3), ineligible subject (*n* = 1), ineligible interventions (*n* = 1) [76], ineligible topic (*n* = 49), not accessible (*n* = 7), and no outcome (*n* = 6). The process of study retrieval and selection is displayed in Fig. 1.

### Characteristics of eligible studies

**Fig. 2:**
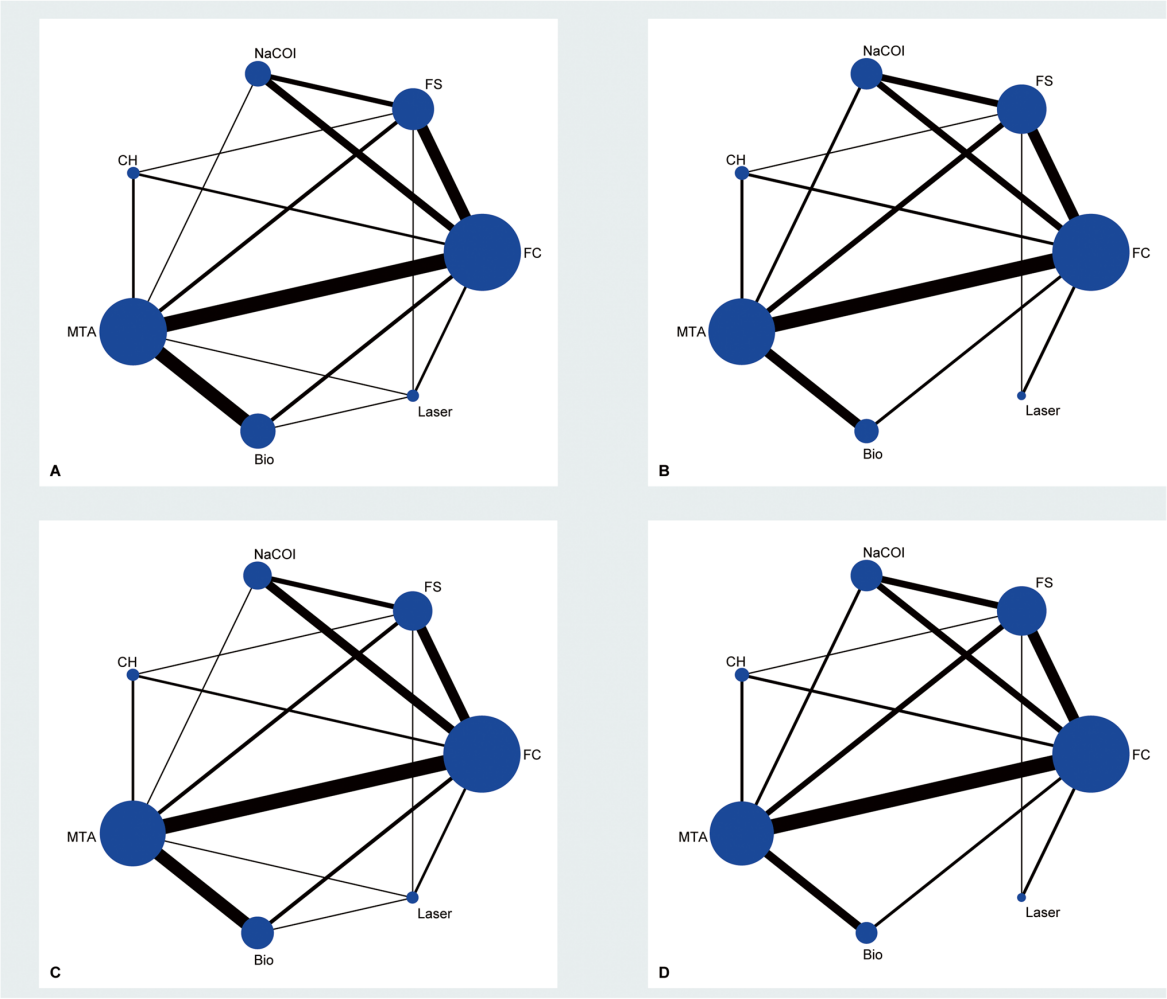
Network plot of clinical success at 6 (**A**) and 12 (**B**) months as well as radiographic success at 6 (**C**) and 12 (**D**) months. The size of an individual node is proportional to the accumulated number of patients, and the thickness of individual line connecting two nodes is proportional to the number of direct comparisons. FC, formocresol; FS, ferric sulfate; NaOCl, sodium hypochlorite; CH, calcium hydroxide; MTA, mineral trioxide aggregate

The characteristics of the eligible studies are presented in Table 1. Among 44 studies included, the follow-up period of individual study was between 6 and 36 months. All studies were published after 2000 except for one study which was published in 1991 [42]. Moreover, the majority of studies were performed in Turkey, Iran, and India. Two-arm design was used in 37 studies [2, 3, 36–39, 42–54, 56, 58–64, 66–75] and three-arm design was used in 6 studies [10, 41, 43, 55, 57, 65]. Forty one studies [2, 3, 10, 36–54, 56–61, 63–75] reported the clinical success rate at 6 months, but only 39 studies [2, 3, 10, 36–43, 45–54, 56–60, 63–75] reported radiographic success rates at 6 months. A total of 36 [2, 3, 36, 37, 39, 41–56, 58, 60–68, 70–73, 75] and 35 [2, 3, 36, 37, 39, 41–56, 58, 60, 62–68, 70–73, 75] studies reported clinical success rate and studies reported radiographic success rate at 12 months. The network plots of different pulpotomy medicaments and techniques for clinical and radiographic success rates at different follow-up durations are displayed in Fig. 2.

**Table 1:** Basic characteristics of included studies in the present network meta-analysis

Author	Country	Comparison	Sample size	Mean age, years	Isolation	Immediate restoration	Final restoration	Follow-up time, months
Ansari, et al. 2018	Iran	15.5% FS	40	4.6 ± 0.6	n.r	reinforced ZOE	SSC	12
		20.0% FC	40					
Erdem, et al. 2011	Turkey	15.5% FS	25	6.16 ± 0.69	Rubber dam	ZOE	SSC	24
		20.0% FC	25					
Farrokh. 2011	Iran	FS	28	6.0 ± 1.6	Rubber dam	ZOE	SSC	9
		FC	24					
Fei, et al. 1991	USA	15.5% FS	29	6.7	Rubber dam	ZOE	SSC	12
		20.0% FC	27					
Huth, et al. 2012	Germany	15.5% FS	50	4.8 ± 1.6	Rubber dam	Reinforced ZOE	SSC	36
		20.0% FC	50					
Ildeş, et al. 2021	USA	20.0% FS	39	6.67 ± 1.08	Rubber dam	Reinforced ZOE	SSC	12
		FC	40	6.83 ± 0.98				
Al-Mutairi, et al. 2013	Saudi Arabia	5% NaOCl	41	5.88 ± 1.29	Rubber dam	Reinforced ZOE	SSC	12
		20.0% FC	41					
Chauhan, et al. 2017	India	5% NaOCl	20	5.9	Rubber dam	ZOE	SSC	6
		20.0% FC	20					
Ruby, et al. 2013	Thailand	3% NaOCl	22	4.6	Rubber dam	ZOE	SSC	12
		20.0% FC	25	5.3				
Shabzendedar, et al. 2013	Iran	3% NaOCl	50	4.3	Rubber dam	Reinforced ZOE	SSC	12
		20.0% FC	50					
Atasever, et al. 2019	Turkey	1.25% NaOCl	40	7.39 ± 1.05	Rubber dam	ZOE	SSC	12
		15.5% FS	40					
Vargas, et al. 2006	Utah	5% NaOCl	32	4–9	Rubber dam	ZOE	SSC	12
		15.5% FS	28					
		NS	30					
Farsi, et al. 2015	Saudi Arabia	5.25% NaOCl	27	7.00 ± 1.40	Rubber dam	ZOE	SSC	18
		15.5% FS	27	6.70 ± 0.98				
		20.0% FC	27	7.50 ± 1.42				
Haideri, et al. 2021	India	20.0% FC	20	n.r	Rubber dam	ZOE	SSC	12
		ProRoot MTA	20					
Cordell, et al. 2021	USA	20.0% FC	25	n.a	Rubber dam	ZOE	SSC	12
		ProRoot MTA	25					
Abuelniel, et al. 2021	Egypt	MTA	30	7.3 ± 1.1	Rubber dam	n.r	ZOE	SSC
		Biodentine	30					
Abd, et al. 2021	Egypt	20.0% FC	24	n.r	Rubber dam	Reinforced ZOE	SSC	12
		MTA	24					
Ramanandvignesh, et al. 2020	India	MTA	18	n.r	Rubber dam	Reinforced ZOE	SSC	9
		Biodentine	18					
		Laser	18					
Pei, et al. 2020	China	20.0% FC	45	4.5 ± 1.2	Rubber dam	Reinforced ZOE	SSC	12
		Laser	45	4.8 ± 1.5				
Alamoudi, et al. 2020	Saudi Arabia	20.0% FC	18	5–8	Rubber dam	Reinforced ZOE	SSC	12
		Laser	18					
Ahuja, et al. 2020	India	20.0% FC	20	4–7	Cotton rolls	ZOE plus zinc phosphate	amalgam	9
		MTA	20					
		Biodentine	20					
Abuelniel, et al. 2020	Egypt	MTA	25	7.5–9	Rubber dam	Reinforced ZOE	SSC	18
		Biodentine	25					
Mythraiye, et al. 2019	India	MTA	28	n.r	Rubber dam	ZOE	SSC	6
		Biodentine	28					
Meligy, et al. 2019	Saudi Arabia	20.0% FC	56	4–8	Rubber dam	Reinforced ZOE	SSC	12
		Biodentine	56					
Çelik, et al. 2019	Turkey	MTA	24	5–9	Rubber dam	Reinforced ZOE	SSC	24
		Biodentine	20					
Nematollahi, et al. 2018	Iran	20.0% FC	25	5–8	Rubber dam	Reinforced ZOE	SSC	24
		MTA	25					
Junqueira, et al. 2018	Brazil	15.5% FS	16	5–9	Rubber dam	Reinforced ZOE	SSC	18
		MTA	15					
Rajasekharan, et al. 2017	Belgium	ProRoot MTA	29	4.6 ± 1.1	Rubber dam	Reinforced ZOE	SSC	12
		Biodentine	25	5.2 ± 1.2				
Juneja, et al. 2017	India	20.0% FC	17	5–9	Rubber dam	Reinforced ZOE	SSC	18
		MTA	17					
		Biodentine	17					
Carti, et al. 2017	Turkey	MTA	25	7.4 ± 1.3	Rubber dam	Reinforced ZOE	SSC	12
		Biodentine	25					
Cuadros, et al. 2016	Spain	MTA	45	4–9	Rubber dam	Reinforced ZOE	SSC	12
		Biodentine	45					
Olatosi, et al. 2016	Nigeria	FC	25	4–7	Rubber dam	Reinforced ZOE	SSC	12
		MTA	25					
Kusum, et al. 2015	India	MTA	25	6.5 ± 1.7	Rubber dam	Reinforced ZOE	SSC	9
		Biodentine	25	6.9 ± 1.7				
Gupta, et al. 2015	India	FS	10	4–10	Rubber dam	reinforced ZOE	SSC	12
		Laser	10					
Oliveira, et al. 2013	Brazil	CH	15	5–9	Rubber dam	Reinforced ZOE	SSC	24
		MTA	15					
Fernández, et al. 2013	Spain	20.0% FS	25	5–9	Rubber dam	Reinforced ZOE	SSC	24
		20.0% FC	25					
		5% NaOCl	25					
		MTA	25					
Sushynski, et al. 2012	USA	FC	114	2.5–10	Rubber dam	Reinforced ZOE	SSC	24
		MTA	108					
Srinivasan, et al. 2011	India	FC	50	4–6	Rubber dam	Reinforced ZOE	SSC	12
		MTA	50					
Lin, et al. 2020	China	15.5% FS	27	2–6	Rubber dam	Reinforced ZOE	SSC	24
		5% NaOCl	27					
		MTA	27					
Zealand, et al. 2010	Canada	FC	103	5.6 ± 1.5	Rubber dam	Reinforced ZOE	SSC	12
		MTA	100					
Alaçam, et al. 2009	Turkey	FC	35	4–8	Rubber dam	Reinforced ZOE	SSC	12
		CH	33					
Sonmez, et al. 2008	Turkey	FS	15	4–9	Rubber dam	Reinforced ZOE	SSC	24
		FC	13					
		CH	13					
		MTA	15					
Noorollahian. 2008	Iran	FC	27	5–7	Rubber dam	Reinforced ZOE	SSC	24
		MTA	29					

*FC*, formocresol; *FS*, ferric sulfate; *NaOCl*, sodium hypochlorite; *CH*, calcium hydroxide; *MTA*, mineral trioxide aggregate; *ZOE*, zinc oxide-eugenol; *RZOE*, reinforced ZOE; *GIC*, glass-ionomer cement; *ZP*, zinc phosphate; *SSC*, stainless steel crown; *n.r.*, not reported; *n.a.*, not available

### Risk of bias of eligible studies

**Fig. 3:**
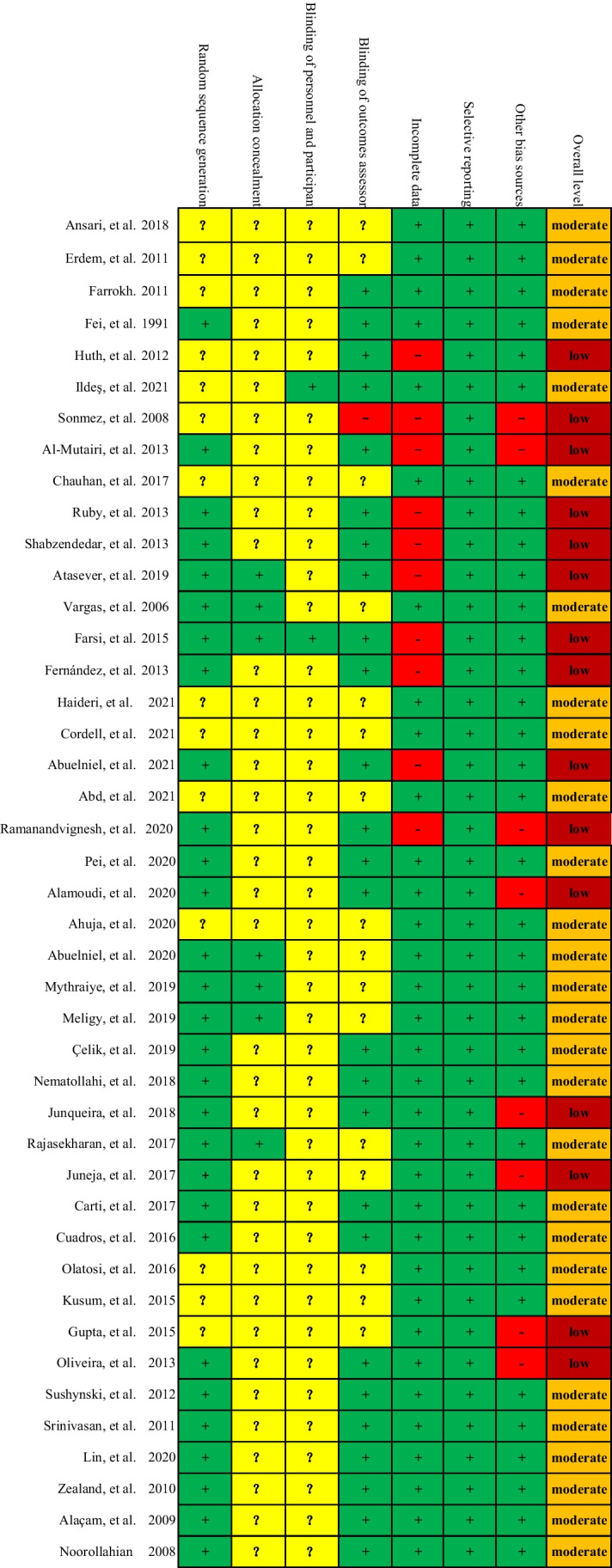
Risk of bias. Green ( +), yellow (?), and red (-) indicates “low,” “unclear,” and “high” risk of bias, respectively

The risk of bias summary is shown in Fig. 3. All studies [2, 3, 10, 36–75] were labeled with “low” or “unclear” risk in random sequence generation, allocation concealment, blinding of participants and personnel, and selective reporting. One study [48] was labeled with “high” risk in blinding of outcomes assessor. Ten studies [36, 37, 41, 43, 44, 46–48, 58, 64] were labeled with “high” risk in incomplete data. Moreover, eight studies [10, 36, 48, 56, 63, 65, 70, 71] were labeled with “high” risk in other sources of risk due to extremely insufficient sample size (< 20 in each group). Overall, 28 studies [3, 36, 38–40, 42, 45, 49–51, 53–55, 57–62, 64, 66–69, 72–75] were rated as “moderate” level, and 15 studies [2, 10, 37, 41, 43, 44, 46–48, 52, 56, 63, 65, 70, 71] were rated as “low” level in the overall methodological quality.

### Inconsistency evaluation

The global inconsistency for individual outcome was evaluated by using the Wald test, and results suggested that the consistency assumption was established for clinical success rates at 6 (*χ*^2^ = 15.57, *P* = 0.555) and 12 (*χ*^2^ = 14.75, *P* = 0.613) months and radiographic success rate at 6 (*χ*^2^ = 24.08, *P* = 0.117) and 12 (*χ*^2^ = 15.29, *P* = 0.574) months. The results of global inconsistency tests are depicted in Fig. S1. Meanwhile, local inconsistency for individual comparison was evaluated by using the node-splitting method, and results suggested no local inconsistency was detected for all comparisons (Table S2). Moreover, loop inconsistency was also checked, and results suggested the absence of loop inconsistency for all outcomes (Table S3).

### Meta-analysis of clinical success rate

**Fig. 4:**
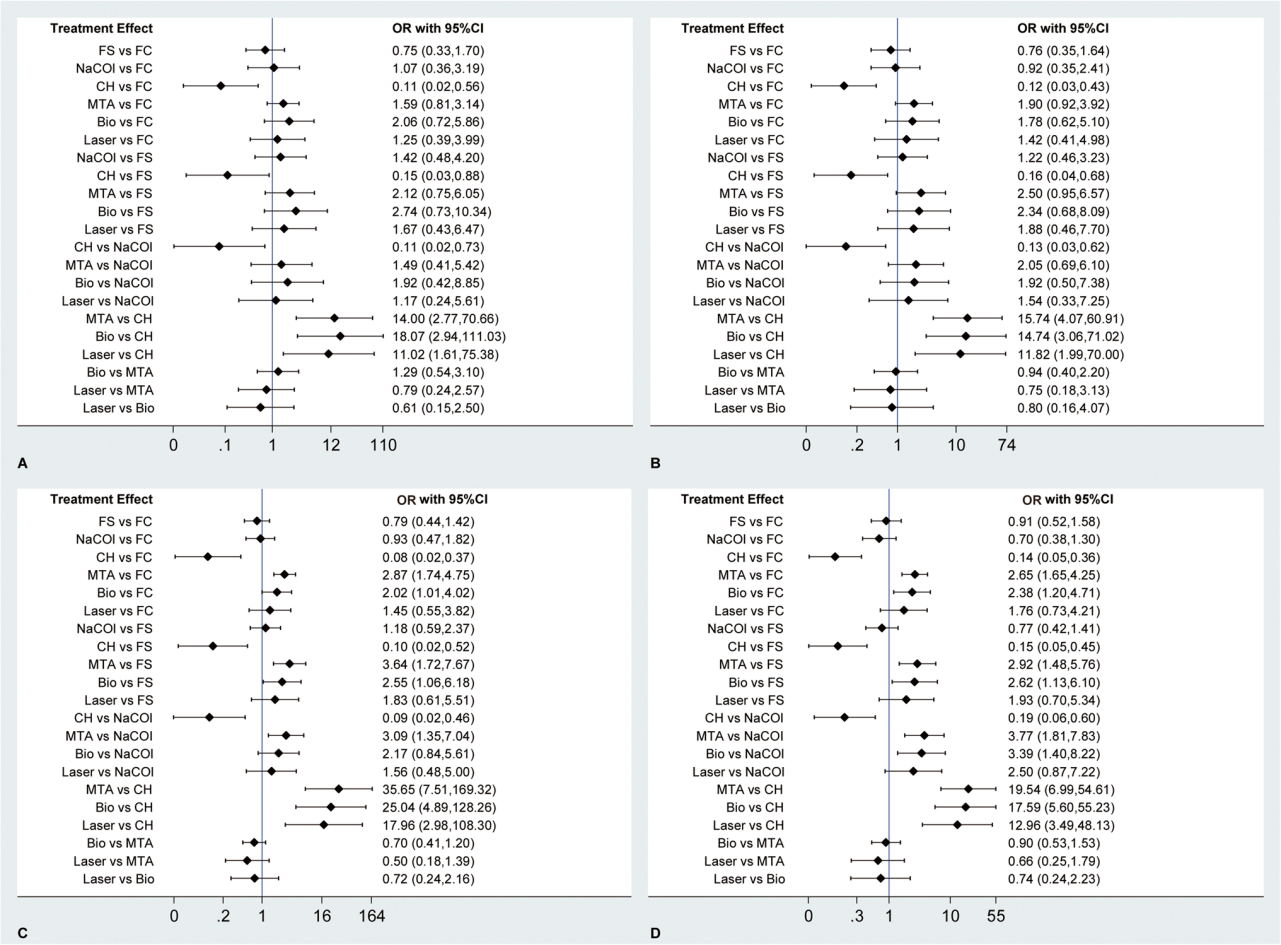
Network meta-analysis of different pulpotomy medicaments for clinical success at 6 (**A**) and 12 (**B**) months as well as radiographic success at 6 (**C**) and 12 (**D**) months. OR, odds ratio; FC, formocresol; FS, ferric sulfate; NaOCl, sodium hypochlorite; CH, calcium hydroxide; MTA, mineral trioxide aggregate

**Fig. 5:**
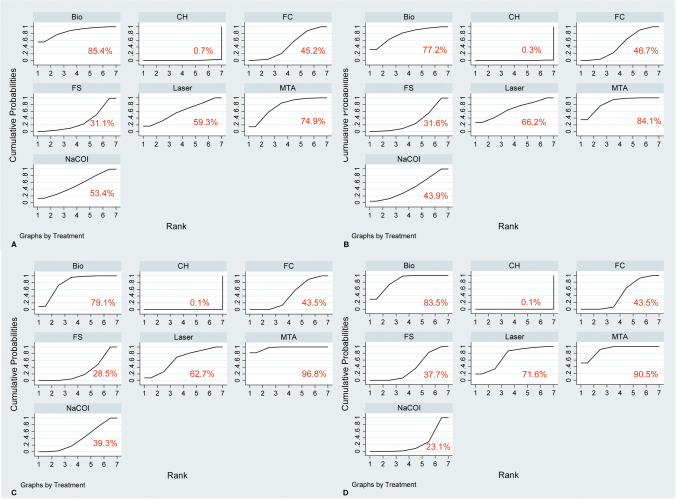
The surface under the cumulative ranking of different pulpotomy medicaments for clinical success at 6 (**A**) and 12 (**B**) months as well as radiographic success at 6 (**C**) and 12 (**D**) months. The red number indicates the numerical value of SUCRA, and a higher SUCRA suggests a higher probability of being a good pulpotomy medicament. FC, formocresol; FS, ferric sulfate; NaOCl, sodium hypochlorite; CH, calcium hydroxide; MTA, mineral trioxide aggregate

Forty-one studies [2, 3, 10, 36–54, 56–61, 63–75] reported clinical success rate at 6 months after pulpotomy treatment, and network meta-analysis suggested that CH was significantly inferior to FC, FS, NaOCl, MTA, biodentine, and laser, but there was no statistical difference for remaining comparisons (Fig. 4A). Moreover, 36 studies [2, 3, 36, 37, 39, 41–56, 58, 60–68, 70–73, 75] reported clinical success rate at 12 months after pulpotomy treatment, and results were not significantly changed (Fig. 4B). Based on the SUCRA method, biodentine ranked first for clinical success rate at 6 months, with a SUCRA value of 85.4%, followed by MTA (74.9%), laser (59.3%), NaCOl (5.4%), FC (45.2%), FS (31.1%), and CH (0.7%) (Fig. 5A); however, MTA had the highest probability of ranking first for clinical success rate at 12 months, with a SUCRA value of 85.4%, followed by biodentine (77.2%), laser (66.2%), FC (46.7%), NaCOl (43.9%), FS (31.6%), and CH (0.3%) (Fig. 5B).

### Meta-analysis of radiographic success rate

A total of 39 eligible studies [2, 3, 10, 36–43, 45–54, 56–60, 63–75] reported radiographic success rate at 6 months after pulpotomy treatment, and network meta-analysis suggested that CH was significantly inferior to FC, FS, NaOCl, MTA, biodentine and laser, and MTA was better than FC, FS, and NaOCl as well as biodentine was better than FC and FS (Fig. 4C). Moreover, 35 studies [2, 3, 36, 37, 39, 41–56, 58, 60, 62–68, 70–73, 75] reported radiographic success rate at 12 months after pulpotomy treatment, and network meta-analysis suggested no significant change in results, except that the comparison of biodentine to NaOCl showed that biodentine was superior to NaCOl in improving radiographic success at 12 months (Fig. 4D). Based on the SUCRA method, MTA had the highest probability of ranking first for radiographic success rate at both 6 and 12 months, with a SUCRA value of 96.8% at 6 months (Fig. 5C) and 90.5% at 12 months (Fig. 5D). Moreover, biodentine and laser had a relatively high probability of becoming second and third for radiographic success rate at both 6 with a SUCRA value of 79.1% and 62.7% (Fig. 5C) and at 12 months with a SUCRA value of 83.5% and 71.6% (Fig. 5D).

### Publication bias

**Fig. 6:**
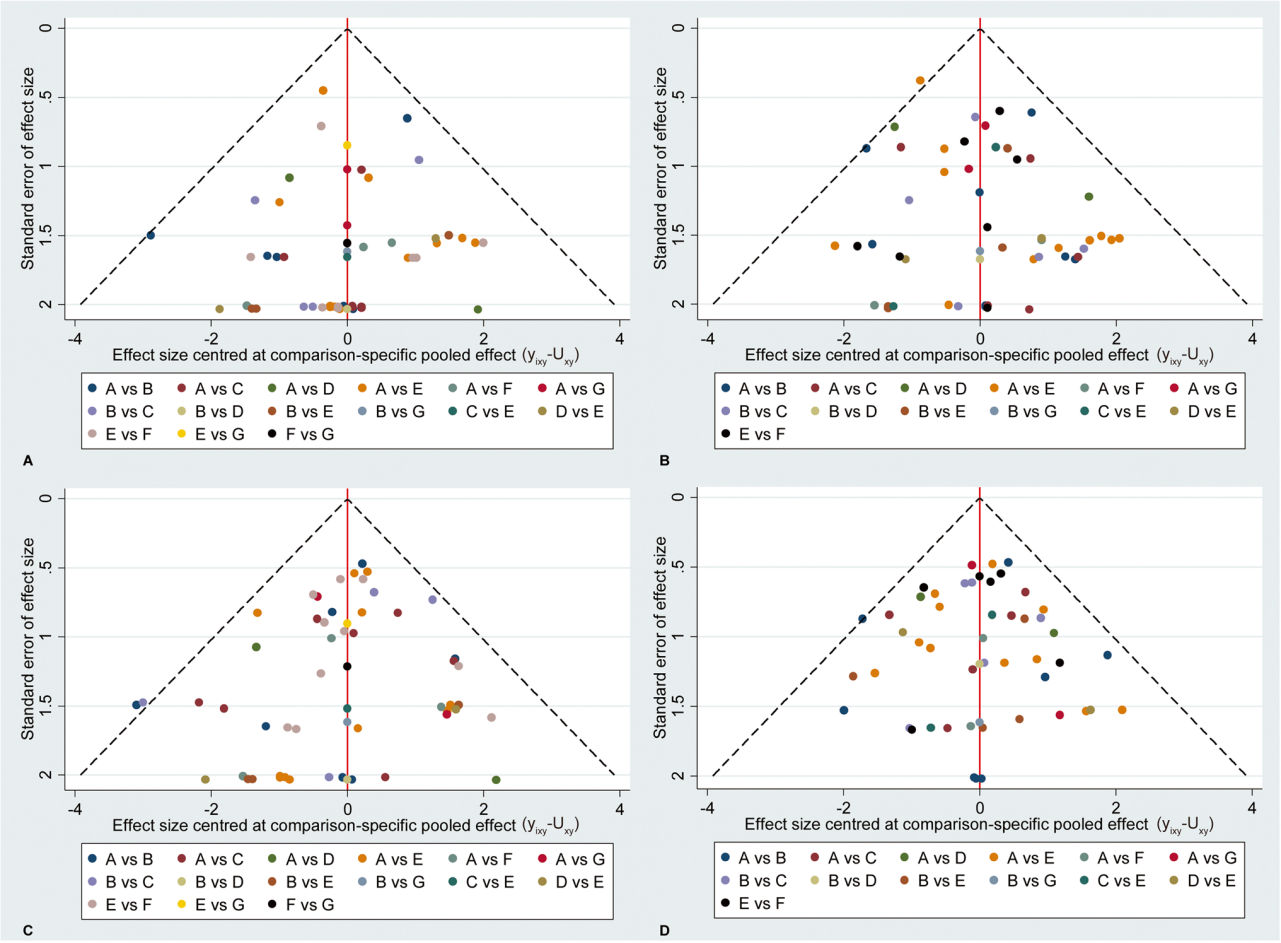
Comparison-adjusted funnel plot for clinical success at 6 (**A**) and 12 (**B**) months as well as radiographic success at 6 (**C**) and 12 (**D**) months. A, B, C, D, E, F, and G represents formocresol, ferric sulfate, sodium hypochlorite, calcium hydroxide, mineral trioxide aggregate, biodentine, and laser, respectively

Comparison-adjusted funnel plot was generated to visually inspect whether presence of publication bias or not for individual outcome. As showed in Fig. 6, the funnel plots suggested that all outcomes could not be negatively influenced.

## Discussion

Pulpotomy continued to be the most widely used endodontic treatment modality in primary dentition [45]. Several pulpotomy medicaments and techniques have also been proposed and used in primary molars, such as FC, FS, MTA, CH, glutaraldehyde (GA), NaOCl, biodentine, lasers and electrosurgery [77]; none are considered ideal [5, 20]. Although previous meta-analyses have compared the efficacy of some pulpotomy medicaments and techniques; however, the optimal option remains unclear due to the comparative success of available medicaments and techniques were not comprehensively evaluated. It is therefore essential to distinguish the comparative efficacy of common pulpotomy medicaments and techniques for primary molars in order to provide a definitive recommendation for clinical decision-making.

In the present network meta-analysis, the comparative success of seven common medicaments and techniques for pulpotomy of primary molars were evaluated in 43 eligible studies, and results suggested CH was inferior to other medicaments and techniques in terms of clinical and radiographic success at both 6 and 12 months. Moreover, MTA was better than FC, FS, and NaOCl, and biodentine was superior to FC and FS. Furthermore, MTA has the highest probability of being optimal option for pulpotomy of primary molars for clinical and radiographic success at both 6 and 12 months, followed by biodentine and laser.

Up to now, several meta-analyses have performed to investigate the comparative efficacy of some pulpotomy medicaments. In meta-analysis by Deery in 2005 [16], 13 studies including three RCTs and 10 clinical trials were included, and pooled result suggested that FC and FS were similar in clinical and radiographic success rates, which were consistent with our pooled results. Meanwhile, meta-analysis [17] by Peng et al. in 2007 also revealed similar clinical and radiographic success between FC and FS in primary molar teeth with exposure of vital pulps by caries or trauma. Recently, an updated meta-analysis with trial sequential analysis was also published [18]. In this meta-analysis of 8 RCTs, authors further suggested that FC and FS showed a comparable clinical and radiographic success at 6, 12, 18, and 24 months. Similarly, network meta-analysis performed by Lin et al. also found comparable efficacy in clinical and radiographic success between FC and FS [20]. Another meta-analysis performed by Nuvvula et al. compared FS with other pulpotomy medicaments [19], but no quantitative synthesis was conducted. Based on available results of the included studies, authors suggested to properly planned RCTs with large sample size and long-term follow-up to further determine the efficacy of FS as an effective pulpotomy medicament. Compared with previous meta-analyses, the present network meta-analysis has three main advantages. First, only RCTs were included in our network meta-analysis, and therefore the risk of introducing bias was significantly reduced. Second, direct and indirect evidence was simultaneously incorporated to estimate the relative efficacy, so all pooled results were more robust and reliable. Third, a total of 43 eligible studies involving 7 common medicaments and techniques were included for data analysis.

Certainly, our network meta-analysis had also several methodological strengths. First, we introduced a comprehensive literature search strategy, which greatly decreased the risk of recall ratio. Second, SUCRA method was introduced to distinguish subtle differences among seven pulpotomy medicaments. Third, we quantify the overall methodological level according to the results of Cochrane risk of bias assessment. Fourth, our network meta-analysis was the first comparison of direct and indirect approaches, which incorporated all available data to evaluate the pulpotomy medicaments more precisely.

Pooled results should also be cautiously interpreted due to several limitations faced by the present network meta-analysis. First, majority of eligible studies included small sample sizes, which may lead to statistical bias. Second, the majority of the included studies were rated to have “low” or “moderate” methodological quality, which may decrease the accuracy of all pooled results. Third, variations were detected in the methods of isolation and restoration and mean age of patients, but subgroup analysis could not be performed due to insufficient data. Fourth, variations were also detected in concentration of FS and NaOCl; our network meta-analysis did not further investigate the comparative efficacy of different concentrations. Therefore, more comprehensive network analysis was needed when sufficient data were available. Fifth, our network meta-analysis only evaluated the clinical and radiographic success at 6 and 12 months. Therefore, long-term efficacy should be further investigated when adequate number of eligible studies were available.

## Conclusion

In conclusion, based on the present study, the results of network meta-analysis revealed CH was the worst medicament and MTA was the best medicament for pulpotomy of primary molars. However, future studies with high quality and large scale are needed to further evaluate the outcomes and consider more medicaments and techniques.

## Supplementary Information

Below is the link to the electronic supplementary material.

Supplementary file1 (PDF 528 KB)

## Data Availability

All data generated or analyzed during this study are included in this published article/as supplementary information files.
