# Supplementary material for: Comparative efficacy of medicaments or techniques for pulpotomy of primary molars: a network meta-analysis
Source: Clin Oral Investig. 2022 Dec 29;27(1):91–104. doi: 10.1007/s00784-022-04830-1 (PMC9876877; doi:10.1007/s00784-022-04830-1)
Supplement: Supplementary file 1 — Supplementary file1 (PDF 528 KB) [file 784_2022_4830_MOESM1_ESM.pdf]

Clinical Oral Investigations

Comparative efficacy of medicaments or techniques for pulpotomy of primary molars: a network meta-analysis

Running title: network meta-analysis of pulpotomy medicaments or techniques

Jiehua Guo[1,*], Na Zhang[1], Yuzhao Cheng[1]

[1]Department of oral medicine, Shanxi Provincial People's Hospital, Taiyuan, Shanxi, China

***Corresponding author**

Jiehua Guo

Department of oral medicine, Shanxi Provincial People's Hospital, NO.29 The twin towers temple District, Taiyuan, Shanxi, China

Email: dentist0355@163.com

Table S1. Detailed search strategies of target databases.
PubMed

No.	Search Details	Results
20	("Pulpotomy"[MeSH Terms] OR ("Pulpotomy"[Title/Abstract] OR "Pulpotomies"[Title/Abstract] OR "dental drilling"[Title/Abstract])) AND ("ferric sulfate"[Supplementary Concept] OR ("ferric sulfate"[Title/Abstract] OR "polyferric sulfate"[Title/Abstract]) OR ("formocresol"[Supplementary Concept] OR ("formocresol"[Title/Abstract] OR "formaldehyde cresol"[Title/Abstract])) OR ("Sodium Hypochlorite"[MeSH Terms] OR ("Sodium Hypochlorite"[Title/Abstract] OR "Antiformin"[Title/Abstract] OR "Clorox"[Title/Abstract])) OR ("Calcium Hydroxide"[MeSH Terms] OR "Calcium Hydroxide"[Title/Abstract]) OR ("MTA"[Title/Abstract] OR (("methods"[MeSH Subheading] OR "methods"[All Fields] OR "mt"[All Fields]) AND "aggregate"[Title/Abstract]) OR "aggregate proroot"[Title/Abstract])) AND ("random*"[All Fields] OR (("allocate"[All Fields] OR "allocated"[All Fields] OR "allocates"[All Fields] OR "allocating"[All Fields] OR "allocation"[All Fields] OR "allocational"[All Fields] OR "allocations"[All Fields] OR "allocative"[All Fields] OR "allocator"[All Fields] OR "allocators"[All Fields]) AND "conceal*"[All Fields]))	267
19	"ferric sulfate"[Supplementary Concept] OR ("ferric sulfate"[Title/Abstract] OR "polyferric sulfate"[Title/Abstract]) OR ("formocresol"[Supplementary Concept] OR ("formocresol"[Title/Abstract] OR "formaldehyde cresol"[Title/Abstract])) OR ("Sodium Hypochlorite"[MeSH Terms] OR ("Sodium Hypochlorite"[Title/Abstract] OR "Antiformin"[Title/Abstract] OR "Clorox"[Title/Abstract])) OR ("Calcium Hydroxide"[MeSH Terms] OR "Calcium Hydroxide"[Title/Abstract]) OR ("MTA"[Title/Abstract] OR (("methods"[MeSH Subheading] OR "methods"[All Fields] OR "mt"[All Fields]) AND "aggregate"[Title/Abstract]) OR "aggregate proroot"[Title/Abstract])	86,702
18	"random*"[All Fields] OR (("allocate"[All Fields] OR "allocated"[All Fields] OR "allocates"[All Fields] OR "allocating"[All Fields] OR "allocation"[All Fields] OR "allocational"[All Fields] OR "allocations"[All Fields] OR "allocative"[All Fields] OR "allocator"[All Fields] OR "allocators"[All Fields]) AND "conceal*"[All Fields])	1,556,941
17	"MTA"[Title/Abstract] OR (("methods"[MeSH Subheading] OR "methods"[All Fields] OR "mt"[All Fields]) AND	20,934

	"aggregate"[Title/Abstract]) OR "aggregate proroot"[Title/Abstract]	
16	"mineral trioxide aggregate"[Supplementary Concept]	1,859
15	"Calcium Hydroxide"[MeSH Terms] OR "Calcium Hydroxide"[Title/Abstract]	6,252
14	"calcium hydroxide"[Title/Abstract]	4,315
13	"Calcium Hydroxide"[MeSH Terms]	4,507
12	"Sodium Hypochlorite"[MeSH Terms] OR "Sodium Hypochlorite"[Title/Abstract] OR "Antiformin"[Title/Abstract] OR "Clorox"[Title/Abstract]	8,629
11	"sodium hypochlorite"[Title/Abstract] OR "Antiformin"[Title/Abstract] OR "Clorox"[Title/Abstract]	6,561
10	"Sodium Hypochlorite"[MeSH Terms]	5,081
9	"formocresol"[Supplementary Concept] OR "formocresol"[Title/Abstract] OR "formaldehyde cresol"[Title/Abstract]	595
8	"formocresol"[Title/Abstract] OR "formaldehyde cresol"[Title/Abstract]	560
7	"formocresol"[Supplementary Concept]	271
6	"ferric sulfate"[Supplementary Concept] OR "ferric sulfate"[Title/Abstract] OR "polyferric sulfate"[Title/Abstract]	626
5	"ferric sulfate"[Title/Abstract] OR "polyferric sulfate"[Title/Abstract]	362
4	"ferric sulfate"[Supplementary Concept]	411
3	"Pulpotomy"[MeSH Terms] OR "Pulpotomy"[Title/Abstract] OR "Pulpotomies"[Title/Abstract] OR "dental drilling"[Title/Abstract]	2,084
2	"Pulpotomy"[Title/Abstract] OR "Pulpotomies"[Title/Abstract] OR "dental drilling"[Title/Abstract]	1,402
1	"Pulpotomy"[MeSH Terms]	1,662

Embase

#No.	Query	Results
#22	#21 AND [embase]/lim	53
#21	#3 AND #19 AND #20	222
#20	random*:ti,ab,kw OR allocation:ti,ab,kw	1843475
#19	#6 OR #9 OR #12 OR #15 OR #18	273028
#18	#16 OR #17	5978
#17	'mineral trioxide aggregate'/exp	1902
#16	'mineral trioxide aggregate':ti,ab,kw OR 'mt aggregate':ti,ab,kw OR mta:ti,ab,kw OR 'aggregate proroot':ti,ab,kw	5849
#15	#13 OR #14	6942

#14	'calcium hydroxide'/exp	6013
#13	'calcium hydroxide':ti,ab,kw	4242
#12	#10 OR #11	10898
#11	'hypochlorite sodium'/exp	9284
#10	'sodium hypochlorite':ti,ab,kw OR antiformin:ti,ab,kw OR clorox:ti,ab,kw	6670
#9	#7 OR #8	682
#8	'formocresol'/exp	574
#7	formocresol:ti,ab,kw OR formacresol:ti,ab,kw OR 'formaldehyde cresol':ti,ab,kw	553
#6	#4 OR #5	864
#5	'ferric sulfate'/exp	722
#4	'ferric sulfate':ti,ab,kw OR 'polyferric sulfate':ti,ab,kw	415
#3	#1 OR #2	1443
#2	'pulpotomy'/exp	493
#1	pulpotomy:ti,ab,kw OR pulpotomies:ti,ab,kw OR 'dental drilling':ti,ab,kw	1362

Cochrane Central Registry of Controlled Trials (CENTRAL)

#No.	Search	Hits
#1	(Pulpotomy):ti,ab,kw OR (Pulpotomies):ti,ab,kw OR (dental drilling):ti,ab,kw	740
#2	MeSH descriptor: [Pulpotomy] explode all trees	183
#3	#1 or #2	740
#4	(ferric sulfate):ti,ab,kw OR (polyferric sulfate):ti,ab,kw	300
#5	(formocresol):ti,ab,kw OR (Formaldehyde cresol):ti,ab,kw	163
#6	(Sodium Hypochlorit):ti,ab,kw OR (Antiformin):ti,ab,kw OR (Clorox):ti,ab,kw	9
#7	MeSH descriptor: [Sodium Hypochlorite] explode all trees	481
#8	#6 or #7	486
#9	(Calcium Hydroxide):ti,ab,kw	869
#10	MeSH descriptor: [Saline Solution] explode all trees	150
#11	#9 or #10	1018
#12	(mineral trioxide aggregate):ti,ab,kw OR (MTA):ti,ab,kw OR (MT aggregate):ti,ab,kw OR (aggregate ProRoot):ti,ab,kw	842
#13	#4 or #5 or #8 or #11 or #12	25483
#14	(random*):ti,ab,kw OR (allocation):ti,ab,kw	1131209
#15	#3 and #13 and #14 in Trials	240

Table S2. Local consistency model test between different pulpotomy medicaments

Local consistency model test was evaluated by using node-splitting method, and a P of >0.05 suggested that local consistency assumption was established. For all outcomes, A, B, C, D, E, F, and G represents formocresol, ferric sulphate, sodium hypochlorite, calcium hydroxide, mineral trioxide aggregate, biodentine, and laser, respectively.

(1) Local inconsistency evaluation for clinical success rate at 6 months.

```
. network sidesplit all, tau
```

Side	Direct		Indirect		Difference			tau
	Coef.	Std. Err.	Coef.	Std. Err.	Coef.	Std. Err.	P>|z|	
A B	-.1448938	.4885681	-.7227344	.8846618	.5778405	1.044539	0.580	.2454361
A C	-.0183985	.6695121	.2638611	1.089181	-.2822596	1.305055	0.829	.2974583
A D	-2.137115	.9880096	-2.231494	1.420251	.094379	1.7256	0.956	.3061202
A E	.3064395	.3837214	1.045369	.7474809	-.7389292	.8585123	0.389	.1963843
A F	1.539784	.923803	.358435	.6147251	1.181349	1.09704	0.282	.1854211
A G	-1.73e-09	.8659389	.439144	.825372	-.439144	1.196281	0.714	.3261899
B C	.816718	.6746899	-.4654708	.8994649	1.282189	1.12579	0.255	.1385903
B D	-.1417319	2.050621	-2.292883	.9881062	2.151152	2.275986	0.345	.2578133
B E	1.984713	.9482562	.2358832	.5341494	1.748829	1.089549	0.108	1.28e-09
B G	1.820713	1.638037	.2378627	.7521797	1.58285	1.802476	0.380	.2652432
C E	.0983934	1.593933	.4743716	.7228101	-.3759782	1.744001	0.829	.3007057
D E	2.157971	1.241473	3.036317	1.125175	-.8783465	1.677681	0.601	.3213794
E F	.1561771	.4584218	1.210116	1.268077	-1.053938	1.313019	0.422	.2324784
E G	-.0695622	.9094762	-.3978202	.8275954	.328258	1.229377	0.789	.33568
F G	-1.494279	1.38956	-.0229183	.9170031	-1.471361	1.752566	0.401	.2840517

(2) Local inconsistency evaluation for clinical success rate at 12 months.

```
. network sidesplit all, tau
```

Side	Direct		Indirect		Difference			tau
	Coef.	Std. Err.	Coef.	Std. Err.	Coef.	Std. Err.	P>|z|	
A B	-.0399655	.4468024	-.9529685	.7738564	.913003	.9003061	0.311	.4421111
A C	-.2556346	.605124	.3072173	.8927584	-.5628519	1.09552	0.607	.511837
A D	-1.983862	.7395351	-2.576135	1.410545	.5922733	1.581983	0.708	.5093049
A E	.4850446	.4183065	1.227591	.8189307	-.742546	.9396481	0.429	.4420701
A F	1.774631	1.222414	.2987236	.5793112	1.475908	1.352262	0.275	.4579616
A G	.1566276	.6897223	1.61303	1.740368	-1.456403	1.872089	0.437	.5032362
B C	.3099784	.5789222	-.1057937	.9666847	.415772	1.117944	0.710	.5085817
B D	-.4560559	1.662982	-2.233605	.8410855	1.777549	1.914949	0.353	.4742363
B E	1.341878	.7173711	.5530696	.6822877	.7888085	1.007149	0.434	.4270769
B G	1.820718	1.692944	.3643106	.7991635	1.456407	1.872082	0.437	.503237
C E	1.27428	.8639416	.3135455	.7473522	.9607349	1.165363	0.410	.4595949
D E	2.098725	1.16089	3.178453	.917214	-1.079728	1.517943	0.477	.5138982
E F	-.1580392	.4454506	1.281838	1.67869	-1.439877	1.72896	0.405	.4695067

(3) Local inconsistency evaluation for radiographic success rate at 6 months.

```
. network sidesplit all, tau
```

Side	Direct Coef.	Direct Std. Err.	Indirect Coef.	Indirect Std. Err.	Difference Coef.	Difference Std. Err.	P>|z|	tau
A B	.0298195	.33974	-1.229361	.6763617	1.25918	.7694598	0.102	9.73e-09
A C	-.5837717	.4075917	1.469841	.7452409	-2.053613	.8806904	0.020	2.02e-10
A D	-2.759601	.9497164	-2.014636	1.374156	-.7449651	1.670409	0.656	.0000433
A E	1.008124	.2896357	1.291833	.6778619	-.2837093	.7481442	0.705	.1091777
A F	1.460964	.7232467	.4671467	.4020001	.9938168	.8272104	0.230	.0001439
A G	.0743807	.64381	.7998022	.7771782	-.7254215	1.009206	0.472	.0000555
B C	.5630904	.4530226	-.4949388	.5824127	1.058029	.7392393	0.152	8.94e-10
B D	-.142912	2.03435	-2.705304	.9043443	2.562392	2.225989	0.250	9.33e-09
B E	1.895256	.8906283	1.162384	.417546	.7328723	.9766618	0.453	1.19e-06
B G	1.820723	1.616419	.4389014	.5988199	1.381821	1.723769	0.423	1.18e-08
C E	1.376	1.235283	1.100716	.4676795	.2752844	1.309549	0.834	.1338581
D E	2.492567	1.219854	4.371361	1.047712	-1.878794	1.607819	0.243	.0000611
E F	-.391641	.2831571	.3471818	1.079906	-.7388228	1.101868	0.503	.1371679
E G	-.0716263	.9027808	-.9855841	.6314818	.9139578	1.101361	0.407	5.46e-08
F G	-.8848115	1.177833	-.1211073	.6976137	-.7637043	1.424977	0.592	.1327275

(4) Local inconsistency evaluation for radiographic success rate at 12 months.

```
. network sidesplit all, tau
```

Side	Direct Coef.	Direct Std. Err.	Indirect Coef.	Indirect Std. Err.	Difference Coef.	Difference Std. Err.	P>|z|	tau
A B	.0947586	.3403871	-.5399608	.5166715	.6347195	.6182941	0.305	9.52e-08
A C	-.6975753	.4016688	.2544287	.5404852	-.952004	.6874748	0.166	3.05e-07
A D	-2.127108	.5744374	-1.540983	1.133086	-.586125	1.300207	0.652	2.11e-08
A E	.8928832	.262613	1.459847	.6677192	-.5669637	.7246615	0.434	3.44e-07
A F	1.335864	.8555848	.7750499	.3811924	.5608141	.9360369	0.549	3.95e-09
A G	.4666433	.4645329	1.760544	1.64199	-1.293901	1.706436	0.448	1.63e-09
B C	-.1033611	.3593907	-.6759079	.5978644	.5725467	.6996158	0.413	7.83e-09
B D	-.9447923	1.12753	-2.287457	.6830956	1.342664	1.374106	0.329	3.02e-10
B E	1.074163	.5892159	1.06804	.421576	.0061231	.7154298	0.993	5.21e-08
B G	1.820739	1.616419	.5268073	.5468818	1.293932	1.706423	0.448	1.03e-09
C E	1.929328	.7063027	1.088458	.4418588	.8408703	.8359243	0.314	1.57e-08
D E	1.826939	.8127069	3.857	.7106158	-2.030061	1.100443	0.065	5.26e-09
E F	-.1542849	.2808196	.742836	1.276168	-.8971209	1.319107	0.496	2.04e-06

Table S3. Loop inconsistency test of first closed loop.

Loop inconsistency was evaluated by using node-splitting method, and loop consistency assumption was established if 95% confidence interval (CI) contained zero. A, B, C, D, E, F, and G represents formocresol, ferric sulphate, sodium hypochlorite, calcium hydroxide, mineral trioxide aggregate, biodentine, and laser, respectively, respectively.

(1) Loop inconsistency evaluation for clinical success rate at 6 months.

Loop	IF	seIF	z_value	p_value	CI_95	Loop_Heterog_tau2
E-F-G	2.179	1.830	1.191	0.234	(0.00,5.77)	0.000
A-B-D	2.070	2.296	0.902	0.367	(0.00,6.57)	0.000
A-B-G	1.870	1.875	0.998	0.318	(0.00,5.54)	0.000
A-E-F	1.217	1.126	1.081	0.280	(0.00,3.42)	0.000
A-B-C	1.214	1.062	1.143	0.253	(0.00,3.30)	0.000
A-B-E	1.125	1.183	0.952	0.341	(0.00,3.44)	0.000
A-F-G	0.728	2.012	0.362	0.717	(0.00,4.67)	0.000
A-C-E	0.669	1.815	0.369	0.712	(0.00,4.23)	0.000
B-C-E	0.582	2.168	0.268	0.788	(0.00,4.83)	0.000
B-E-G	0.551	2.098	0.263	0.793	(0.00,4.66)	0.000
A-D-E	0.250	1.583	0.158	0.874	(0.00,3.35)	0.000
B-D-E	0.220	2.660	0.083	0.934	(0.00,5.43)	0.000
A-E-G	0.183	1.230	0.149	0.882	(0.00,2.59)	0.000

(2) Loop inconsistency evaluation for clinical success rate at 12 months.

Loop	IF	seIF	z_value	p_value	CI_95	Loop_Heterog_tau2
A-B-G	1.602	1.763	0.909	0.364	(0.00,5.06)	0.000
A-E-F	1.572	1.310	1.200	0.230	(0.00,4.14)	0.000
A-B-C	1.132	0.883	1.282	0.200	(0.00,2.86)	0.000
A-B-E	0.719	0.966	0.745	0.456	(0.00,2.61)	0.253
B-D-E	0.421	2.143	0.197	0.844	(0.00,4.62)	0.000
B-C-E	0.410	1.341	0.306	0.760	(0.00,3.04)	0.000
A-C-E	0.404	1.174	0.344	0.731	(0.00,2.71)	0.286
A-B-D	0.264	2.158	0.122	0.903	(0.00,4.49)	0.727
A-D-E	0.049	1.621	0.030	0.976	(0.00,3.23)	0.707

(3) Loop inconsistency evaluation for radiographic success rate at 6 months.

Loop	IF	seIF	z_value	p_value	CI_95	Loop_Heterog_tau2
A-B-D	2.422	2.541	0.953	0.340	(0.00,7.40)	0.424
A-B-G	1.784	1.798	0.992	0.321	(0.00,5.31)	0.035
E-F-G	1.728	1.540	1.122	0.262	(0.00,4.75)	0.000
A-B-C	1.364	0.871	1.566	0.117	(0.00,3.07)	0.230
A-D-E	1.272	1.572	0.809	0.419	(0.00,4.35)	0.000
A-E-F	1.037	0.880	1.178	0.239	(0.00,2.76)	0.000
B-C-E	1.004	2.984	0.336	0.737	(0.00,6.85)	2.917
A-E-G	0.839	1.145	0.733	0.464	(0.00,3.08)	0.000
A-C-E	0.819	1.602	0.511	0.609	(0.00,3.96)	0.000
B-E-G	0.498	2.287	0.218	0.828	(0.00,4.98)	0.310
A-B-E	0.484	1.129	0.429	0.668	(0.00,2.70)	0.000
B-D-E	0.409	2.659	0.154	0.878	(0.00,5.62)	0.000
A-F-G	0.151	1.577	0.096	0.924	(0.00,3.24)	0.000

(4) Loop inconsistency evaluation for radiographic success rate at 12 months.

Loop	IF	seIF	z_value	p_value	CI_95	Loop_Heterog_tau2
B-D-E	1.603	1.607	0.998	0.318	(0.00,4.75)	0.000
A-B-G	1.222	1.916	0.638	0.524	(0.00,4.98)	0.359
A-D-E	1.136	1.176	0.966	0.334	(0.00,3.44)	0.202
A-B-C	1.058	0.665	1.592	0.111	(0.00,2.36)	0.000
B-C-E	0.812	1.079	0.753	0.452	(0.00,2.93)	0.000
A-E-F	0.566	0.945	0.599	0.549	(0.00,2.42)	0.000
A-C-E	0.191	0.897	0.212	0.832	(0.00,1.95)	0.000
A-B-E	0.059	0.821	0.072	0.943	(0.00,1.67)	0.131
A-B-D	0.016	1.807	0.009	0.993	(0.00,3.56)	0.774

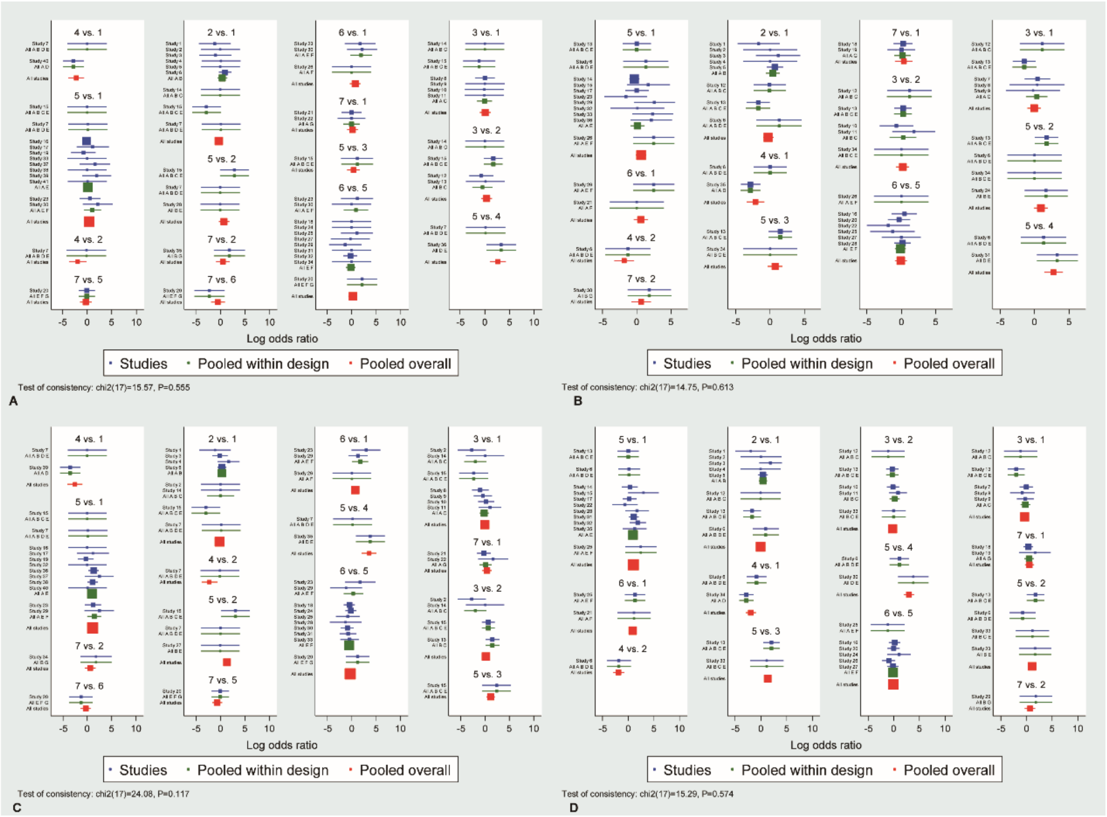

Figure S1. Global inconsistency assessment of clinical and radiographic success at 6 and 12 months. In this figures, 1 to 7 represents formocresol, ferric sulphate, sodium hypochlorite, calcium hydroxide, mineral trioxide aggregate, biodentine, and laser, respectively, respectively.
